# Serotype-Specific Biochemical and Immunological Signatures of Dengue Virus Envelope Proteins

**DOI:** 10.3390/cimb48060631

**Published:** 2026-06-17

**Authors:** Iasmin V. Costa, Ana Cecília R. Cruz, Carlos Alberto M. Carvalho

**Affiliations:** 1 Laboratory of Molecular Biology, Center for Biological and Health Sciences, University of Pará State, Belém 66095-662, PA, Brazil; iasmin.costa@aluno.uepa.br; 2 Laboratory of Molecular Biology, Section for Arbovirology and Hemorrhagic Fevers, Evandro Chagas Institute, Ananindeua 67030-000, PA, Brazil; anacecilia@iec.gov.br

**Keywords:** dengue virus, computational biology, viral fusion proteins

## Abstract

Dengue is an arboviral disease of global significance caused by *Orthoflavivirus denguei* (DENV), which has four antigenically distinct serotypes. The envelope (E) protein plays a critical role in viral entry and eliciting immune responses. This study aimed to compare the biochemical and immunological properties of the E protein across the four DENV serotypes using in silico approaches. E protein reference sequences were retrieved from RefSeq and analyzed with various bioinformatics tools. Sequence alignment revealed identities ranging from 63.08% to 77.69%. Biochemical analysis showed minimal variation in molecular weight and isoelectric point; however, the net charge of DENV-3 E protein was notably lower. Secondary structure predictions indicated a predominance of alpha-helices in DENVs-1/2, while DENVs-3/4 featured more beta-sheets. Post-translational modification analysis revealed mostly casein kinase II phosphorylation sites across all serotypes, with DENV-4 uniquely presenting also tyrosine kinase sites. Amino acids W231/D341 in DENV-1, Q86 in DENVs-2/4, and D87/D339 in DENV-3 showed maximum antigenicity scores in B cell recognition, while the human leukocyte antigen (HLA) alleles B*08:01/B*39:01 and DRB4*01:01, recognized by T cells, presented the highest number of predicted epitopes for the different DENV serotypes. Conservation analysis showed that the major antigenic regions highlighted in this study are highly conserved among contemporary DENV isolates despite the genetic variability observed within each serotype. These findings suggest that subtle structural differences in the E protein may contribute to distinct immunogenic profiles, highlighting candidate regions for future investigation.

## 1. Introduction

Dengue is a mosquito-borne viral disease caused by *Orthoflavivirus denguei* (DENV), a member of the *Flaviviridae* family represented by four distinct serotypes (DENV-1 to DENV-4). Like other members of this family, DENV is transmitted by hematophagous arthropods, primarily *Aedes aegypti*, and can lead to clinical manifestations ranging from asymptomatic infections to severe conditions such as dengue hemorrhagic fever (DHF) and dengue shock syndrome (DSS) [1].

Globally, dengue poses a significant public health burden, particularly in tropical and subtropical regions. It is estimated that approximately 390 million dengue infections occur annually, with up to 1 million classified as severe cases. Climate change and anthropogenic pressure on ecosystems have raised concerns about the spread of the disease to new areas as well as increased severity in endemic zones [2].

The positive-sense, single-stranded RNA genome of DENV encodes a polyprotein that is cleaved into three structural proteins—C (capsid), prM/M (pre-membrane/membrane), and E (envelope)—and seven non-structural proteins (NS1, NS2A, NS2B, NS3, NS4A, NS4B, and NS5). E protein is essential for viral entry, mediating attachment and fusion with host cell membranes via conformational changes triggered by endosomal acidification [3]. This protein is also the primary target of neutralizing antibodies, making it a critical element for understanding the immune response to DENV infection. Although the four DENV serotypes share high sequence similarity, subtle amino acid variations can significantly impact their antigenicity and pathogenicity [4]. Previous computational investigations have explored distinct aspects of DENV E protein, including structural modeling, post-translational modification analyses, and epitope prediction [5,6,7]. However, these studies generally focused on specific serotypes, selected epitopes, or individual analytical approaches rather than providing an integrated comparison across all four DENV serotypes.

To address this gap, the present study combined multiple bioinformatics approaches within a unified comparative framework to evaluate E protein reference sequences from the four DENV serotypes. Specifically, we sought to identify key amino acid substitutions and determine molecular weight, net charge, isoelectric point, and residual polarity. We also analyzed the secondary structure, predicted post-translational modifications, and evaluated the antigenicity of the protein based on hydrophilicity scores. Finally, we predicted the interaction affinity of E protein-derived peptides with both classes of human leukocyte antigens (HLA), aiming to identify conserved and immunodominant epitopes. Because a single reference sequence was selected for each serotype, the results should be interpreted as a comparative assessment of representative genomes rather than a comprehensive characterization of the genetic diversity present within circulating DENV populations.

## 2. Materials and Methods

### 2.1. Sequence Retrieval

Nucleotide sequences coding for the E protein from the four DENV serotypes were retrieved from reference sequences of the complete viral genomes (DENV-1: NC_001477.1, DENV-2: NC_001474.2, DENV-3: NC_001475.2, DENV-4: NC_002640.1) available on the NCBI RefSeq database [8]. These sequences were conceptually translated into amino acid sequences using the EMBOSS Transeq 6.6.0 (EMBL’s European Bioinformatics Institute, Hinxton, UK) tool [9], applying the standard code as codon table, and saved in FASTA format. In compliance with Brazilian Law No. 13,123/2015 and its regulatory decrees, this research was registered in the National System for the Management of Genetic Heritage and Associated Traditional Knowledge (SisGen) under the number AC4C6ED. The use of a single RefSeq accession per serotype was intended to provide a standardized comparative framework across the four DENV serotypes.

### 2.2. Multiple Sequence Alignment

The amino acid sequences of the E protein from the four DENV serotypes were aligned on Clustal Omega 1.2.4 (EMBL’s European Bioinformatics Institute, Hinxton, UK) [10], a program that uses seeded guide trees and hidden Markov models to determine conserved and variable regions, with default parameters. A percent identity matrix was generated from the alignment, and amino acid substitutions identified from the multiple sequence alignment were classified according to the degree of physicochemical similarity between aligned residues, based on the similarity scores derived from the PAM 250 substitution matrix [11]. Conservative substitutions were defined as residue replacements exhibiting similarity scores greater than 0.5, semi-conservative substitutions as replacements with similarity scores greater than 0 and up to 0.5, and non-conservative substitutions as replacements with similarity scores equal to or below 0.

### 2.3. Sequence Statistics

Molecular weight, isoelectric point, net charge, and side-chain polarity of the E protein from each DENV serotype were estimated using the EMBOSS Pepstats 6.6.0 (EMBL’s European Bioinformatics Institute, Hinxton, UK) tool [9], configured to include termini charges and not use monoisotopic weights. Side chain polarity was classified into four categories: nonpolar, polar neutral, polar acidic, and polar basic.

### 2.4. Prediction of Secondary Structures

Secondary structures of DENV-1 to DENV-4 E proteins were predicted using the NPS@ Predator (IBPC’s Rhone Alpes Bioinformatic Pole, Lyon, France) web server [12], which recognizes potentially hydrogen-bonded residues in a single amino acid sequence to identify alpha helices, beta sheets, and random coils, along with their positions in the polypeptide chains. Output width was set to 70 residues and secondary structure data to DSSP.

### 2.5. Identification of Potential Post-Translational Modifications

Potential post-translational modification sites in the E protein from each DENV serotype were identified using the NPS@ Proscan (IBPC’s Rhone Alpes Bioinformatic Pole, Lyon, France) web server [12]. This tool scans a sequence against the PROSITE database in search of consensus patterns related to common post-translational modifications such as glycosylation, myristoylation, and phosphorylation. The similarity level was defined as no mismatch (i.e., 100% similarity).

### 2.6. Prediction of B-Cell Epitopes

B-cell epitopes in DENV-1 to DENV-4 E proteins were computed using the NPS@ PCProf (IBPC’s Rhone Alpes Bioinformatic Pole, Lyon, France) web server [11], which uses a hydrophilicity scale derived from high-performance liquid chromatography peptide retention data to predict surface residues with antigenicity [13]. The analysis was conducted using a window size of 7 residues. Because this approach is sequence-based, the analysis is restricted to the identification of potential linear epitopes and does not account for conformational epitopes arising from the three-dimensional organization of the protein.

### 2.7. Prediction of T-Cell Epitopes

To assess potential epitopes of T CD4^+^ and T CD8^+^ cells, E protein sequences from the four DENV serotypes were submitted to the NetMHCpan-4.1 and NetMHCIIpan-4.0 (DTU’s Department of Health Technology, Kongens Lyngby, Denmark) servers [14], respectively. For HLA-I, representative alleles from major supertypes (A*01:01, A*02:01, A*03:01, A*24:02, A*26:01, B*07:02, B*08:01, B*15:01, B*27:05, B*39:01, B*40:01, and B*58:01) were used for 9-mer peptides. For HLA-II, the 7-allele (DRB1*03:01, DRB1*07:01, DRB1*15:01, DRB3*01:01, DRB3*02:02, DRB4*01:01, and DRB5*01:01) reference method [15] was employed for 15-mer peptides. The objective was to achieve broad functional coverage rather than represent the frequency distribution of HLA alleles in a specific population. For an in-depth analysis of predicted epitopes, results were processed using the Epitope-Evaluator (BU’s Fuxman Bass Lab, Boston, MA, USA) tool [16], considering affinity thresholds of 2% and 5% rank for peptides binding to HLA-I and HLA-II alleles, respectively, which correspond to the default criteria for identifying high-affinity binders in NetMHCpan and NetMHCIIpan predictions, prioritizing peptides with the greatest likelihood of HLA presentation.

### 2.8. Conservation Analysis of Predicted Epitopes

To evaluate whether the predicted immunologically relevant regions identified in the reference sequences were conserved among circulating DENV strains, additional E protein sequences were retrieved from the NCBI GenBank database [17]. For each serotype, the most recent sequences available from human blood isolates representing five continents were selected as follows: AIX94452.1 (Angola, 2013), BBH51260.1 (Bangladesh, 2017), QBF29196.1 (USA, 2015), QBM19392.1 (French Polynesia, 2018), and QBA18566.1 (Brazil, 2016) for DENV-1; QCT81069.1 (Côte d’Ivoire, 2017), BBH51262.1 (Bangladesh, 2017), AIW42845.1 (Puerto Rico, 2013), QBM19388.1 (French Polynesia, 2018), and AEN71241.1 (Colombia, 2007) for DENV-2; AGR44695.1 (Somalia, 2011), BBH51309.1 (Bangladesh, 2017), AIZ00463.1 (USA, 2014), AIW42835.1 (Fiji, 2014), and AEN71254.1 (Colombia, 2007) for DENV-3; and AYP74651.1 (China, 2017), ANC57624.1 (Italy, 2015), QBF29232.1 (Puerto Rico, 2014), AFZ89024.1 (Micronesia, 2012), and ANC57627.1 (Brazil, 2013) for DENV-4. Consensus sequences were generated using the EMBOSS Cons 6.6.0 (EMBL’s European Bioinformatics Institute, Hinxton, UK) tool [9] and subsequently aligned with their corresponding reference sequences using the EMBOSS Needle 6.6.0 (EMBL’s European Bioinformatics Institute, Hinxton, UK) tool [9]. The conservation of prominent B-cell epitopes and promiscuous T-cell epitopes identified in this study was then assessed by comparing their amino acid sequences between the consensus and reference sequences.

## 3. Results

### 3.1. Sequence Identity Among DENV E Proteins

**Figure 1:**
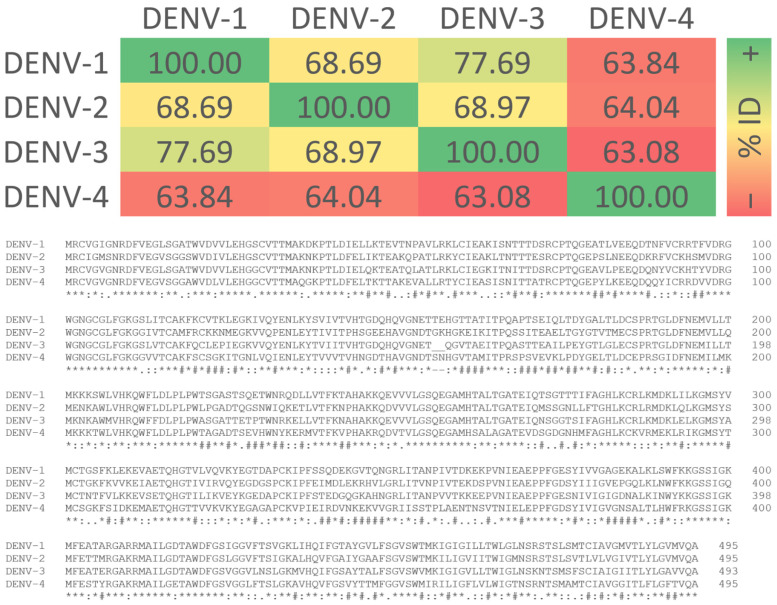
Percent identity matrix and classification of mutations of E protein sequences across the four DENV serotypes. A multiple sequence alignment of the query proteins was performed to generate a percent identity matrix of their constituent residues (**top**), visualized using a red–yellow–green gradient to indicate increasing identity (ID) values. Mutations were classified from the multiple sequence alignment based on the physicochemical properties of the involved residues (**bottom**), so that asterisks (*) represent positions where all aligned sequences have the same residue, colons (:) indicate positions where residues have strongly similar properties (i.e., conservative substitutions), periods (.) represent positions where residues have weakly similar properties (i.e., semi-conservative substitutions), hashes (#) indicate positions where residues are not similar (i.e., non-conserved substitutions), and dashes (–) mean positions where at least one aligned sequence has a residue insertion or deletion.

The multiple sequence alignment of E protein amino acid sequences from the four DENV serotypes revealed percent identity values ranging from 63.08% (between DENV-3 and DENV-4) to 77.69% (between DENV-1 and DENV-3). Most of the 233 amino acid mismatches found among the polypeptide chains corresponded to substitutions with a conservative character regarding residual properties (45.49%); semi-conservative and non-conservative substitutions represented 15.02% and 38.63%, respectively, while insertions/deletions comprised 0.86% of such mismatches (Figure 1).

### 3.2. Residual Properties of DENV E Proteins

The E protein molecular weights ranged from 53.68 kDa (DENV-3) to 54.44 kDa (DENV-2), while isoelectric points varied between 7.01 (DENV-3) and 7.89 (DENV-1). The net charge showed greater disparity, with DENV-3 presenting the lowest value (3.50) and DENV-4 the highest (8.50) at pH 7.0 (Table 1).

**Figure 2:**
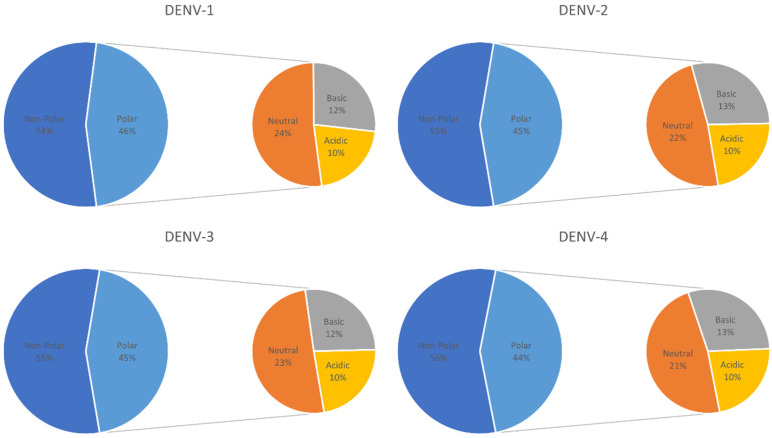
Side-chain polarity distributions of E proteins from DENV-1 to DENV-4. Residues were grouped according to their polarity as non-polar (A, C, F, G, I, L, M, P, V, W, and Y) or polar (D, E, H, K, N, Q, R, S, and T), with polar residues further classified as neutral (N, Q, S, and T), basic (H, K, and R), or acidic (D and E) based on their charge.

In all DENV serotypes, the E protein showed a predominance of non-polar residues (54–56%). Among polar residues (44–46%), DENV-1 and DENV-3 presented a slight predominance of neutral over charged (basic and acidic) residues (23–24% vs. 22%, respectively), whereas DENV-2 and DENV-4 showed the inverse distribution (21–22% vs. 23%, respectively) (Figure 2).

### 3.3. Secondary Structure Content in DENV E Proteins

**Figure 3:**
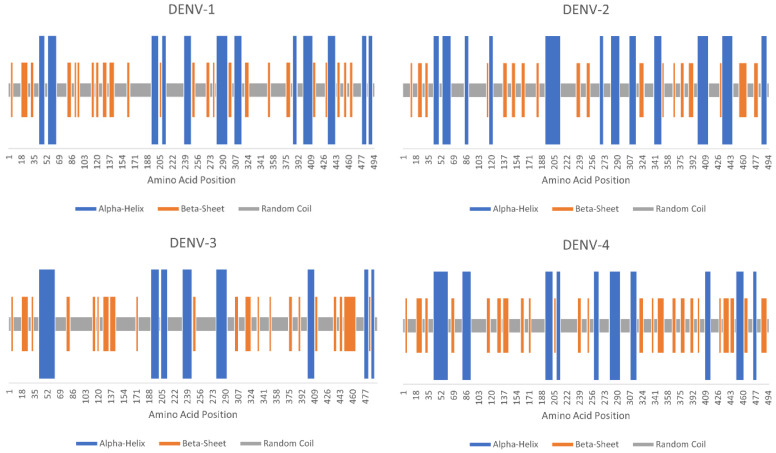
Predicted secondary structures along E proteins from the four DENV serotypes. Residues were assigned to alpha-helices, beta-sheets, or random coils based on long-range hydrogen bonding patterns, with their locations identified along the polypeptide chains.

Random coils predominated in all four DENV serotypes, ranging from 53.74% (DENV-4) to 59.63% (DENV-3). DENV-1 and DENV-2 exhibited a higher predicted proportion of alpha-helices than beta-sheets (23.03% vs. 21.82% and 25.86% vs. 18.59%, respectively). In contrast, DENV-3 and DENV-4 showed more beta-sheets than alpha-helices (21.91% vs. 18.46% and 25.25% vs. 21.01%, respectively) (Figure 3).

### 3.4. Potential Post-Translational Modifications in DENV E Proteins

**Figure 4:**
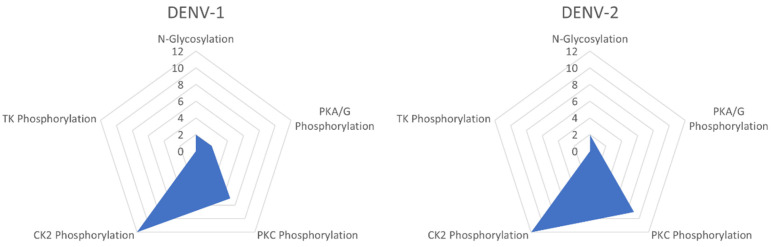

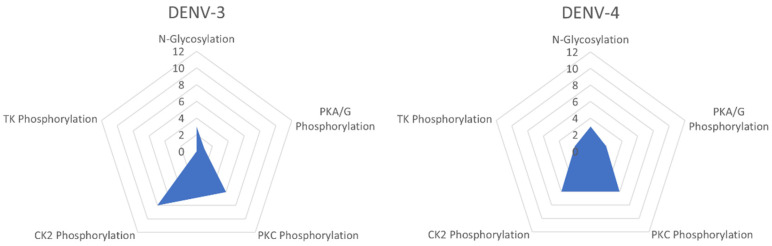
Predicted post-translational modifications in E proteins from DENV-1 to DENV-4. Amino acid sequences were scanned for several consensus patterns, including N-glycosylation (N–{P}–[ST]–{P}), PKA/G phosphorylation ([RK](2)–X–[ST]), PKC phosphorylation ([ST]–X–[RK]), CK2 phosphorylation ([ST]–X(2)–[DE]), and TK phosphorylation ([RK]–X(2,3)–[DE]–X(2,3)–Y), whose occurrences were plotted as absolute numbers.

Consensus patterns for several post-translational modifications were present across all DENV serotypes, including N-glycosylation and phosphorylations by cAMP- and cGMP-dependent protein kinase (PKA/G), protein kinase C (PKC), casein kinase II (CK2), and tyrosine kinase (TK). Notably, most of the consensus patterns present in the E protein of the four DENV serotypes corresponded to CK2 phosphorylation (6–12 sites). TK phosphorylation sites were exclusive to DENV-4, with two predicted sites: KEEQDQQY (83–90) and KMFESTY (400–406). Other consensus patterns included N-glycosylation (2–3 sites), PKA/G phosphorylation (1–2 sites), and PKC phosphorylation (6–9 sites) (Figure 4).

### 3.5. B-Cell Epitopes on DENV E Proteins

**Figure 5:**
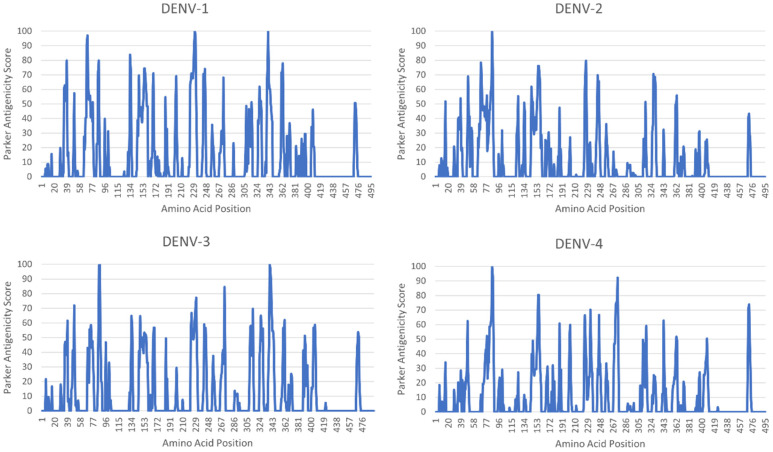
Potential linear epitopes on E proteins from DENV-1 to DENV-4. Proteins were evaluated for antigenic propensity based on a hydrophilicity scale, expressed as scores along amino acid positions in the polypeptide chains.

The hydrophilicity scores of the residues revealed a higher antigenic propensity in the N-terminal half of the E protein of the four DENV serotypes, although a characteristic profile was observed for DENV-1 and DENV-3 and another for DENV-2 and DENV-4. The epitopes around amino acids W231/D341 in DENV-1, Q86 in DENV-2 and DENV-4, and D87/D339 in DENV-3 showed maximum predicted antigenicity in the recognition by B cells (Figure 5).

### 3.6. T-Cell Epitopes in DENV E Proteins

**Figure 6:**
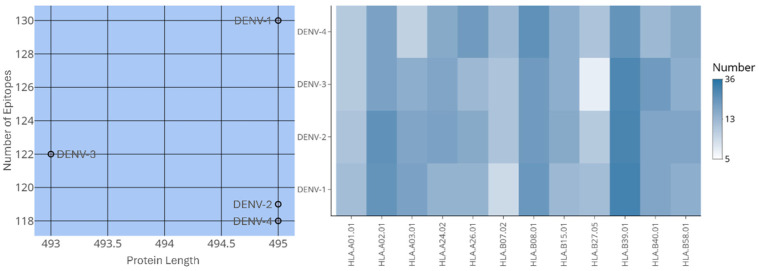
Number of predicted epitopes in E proteins from the four DENV serotypes for HLA-I. (**On the left**), correlation between the number of 9-mer epitopes and the length of the E protein. (**On the right**), correlation between the number of 9-mer epitopes and the HLA-I allele for each DENV serotype.

Regarding HLA-I alleles, DENV-1 presented the highest number of epitopes (130 epitopes), while DENV-4 had the lowest number (118 epitopes), with DENV-2 and DENV-3 having intermediate numbers (119 and 122 epitopes, respectively). B*39:01 was the allele with the highest number of predicted epitopes for DENV-1 (29 epitopes), DENV-2 (28 epitopes) and DENV-3 (27 epitopes), with this position being occupied by the B*08:01 allele for DENV-4 (23 epitopes) (Figure 6).

**Figure 7:**
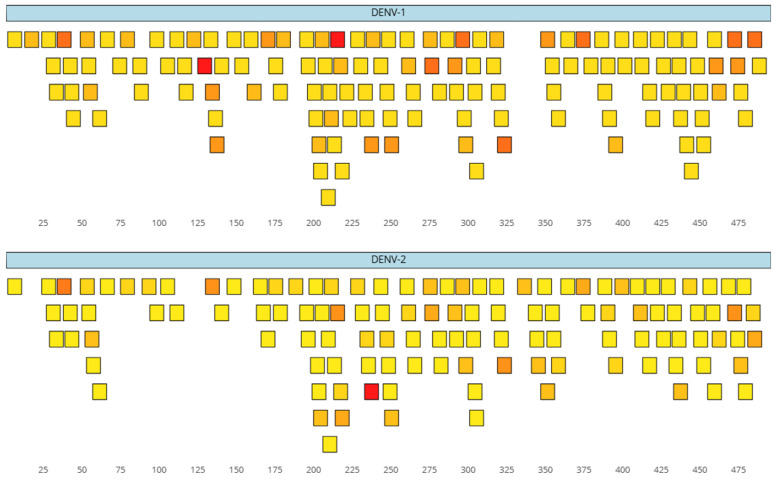

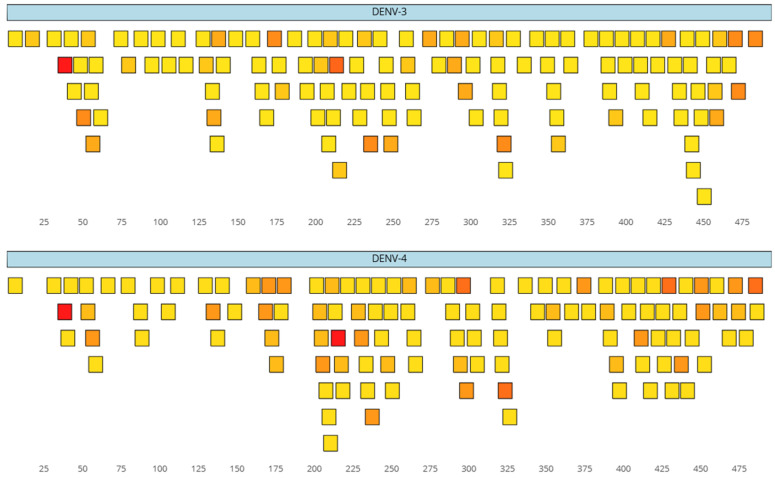
Location of predicted epitopes in E proteins from the four DENV serotypes for HLA-I. The E protein of each DENV serotype is displayed as a light blue bar, while predicted 9-mer epitopes are represented in a color gradient from yellow to red, indicating the number of HLA-I alleles to which each epitope can bind and ranging from 1 to 5 alleles for DENV-1 and DENV-4, from 1 to 8 alleles for DENV-2, and from 1 to 6 alleles for DENV-3. Regions in red thus indicate promiscuous epitopes, recognized by a greater number of alleles. Below each panel, the position of the amino acids in the polypeptide chain is reported.

Still regarding HLA-I alleles, it was observed that epitopes with high affinity for multiple of them were frequently located in divergent regions of the E protein of each DENV serotype (Figure 7). For DENV-1, the epitopes KLEGKIVQY (124–132) and KQWFLDLPL (210–218) were able to bind to 5 alleles (A*01:01/A*03:01/A*26:01/B*15:01/B*58:01 and A*02:01/B*15:01/B*27:05/B*39:01/B*40:01, respectively). For DENV-2, the epitope IQKETLVTF (232–240) was able to bind to 8 alleles (A*24:02/A*26:01/B*08:01/B*15:01/B*27:05/B*39:01/B*40:01/B*58:01). For DENV-3, the epitope TMAKNKPTL (33–41) was able to bind to 6 alleles (A*02:01/A*24:02/B*07:02/B*08:01/B*15:01/B*39:01). For DENV-4, the epitopes TMAQGKPTL (33–41) and KQWFLDLPL (210–218) were able to bind to 5 alleles (A*02:01/B*07:02/B*08:01/B*15:01/B*39:01 and A*02:01/B*15:01/B*27:05/B*39:01/B*40:01, respectively).

**Figure 8:**
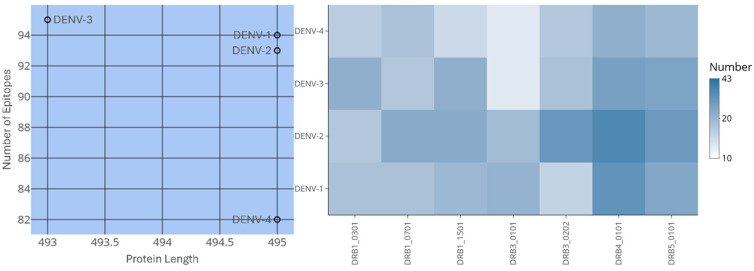
Number of predicted epitopes in E proteins from the four DENV serotypes for HLA-II. (**On the left**), correlation between number of 15-mer epitopes and length of the E protein. (**On the right**), correlation between number of 15-mer epitopes and HLA-II allele for each DENV serotype.

Regarding HLA-II alleles, DENV-3 presented the highest number of epitopes (95 epitopes), while DENV-4 had the lowest number (82 epitopes), with DENV-1 and DENV-2 having intermediate numbers (94 and 93 epitopes, respectively). DRB4*01:01 was the allele with the highest number of predicted epitopes for DENV-1 (30 epitopes), DENV-2 (34 epitopes), DENV-3 (26 epitopes) and DENV-4 (22 epitopes) (Figure 8).

**Figure 9:**
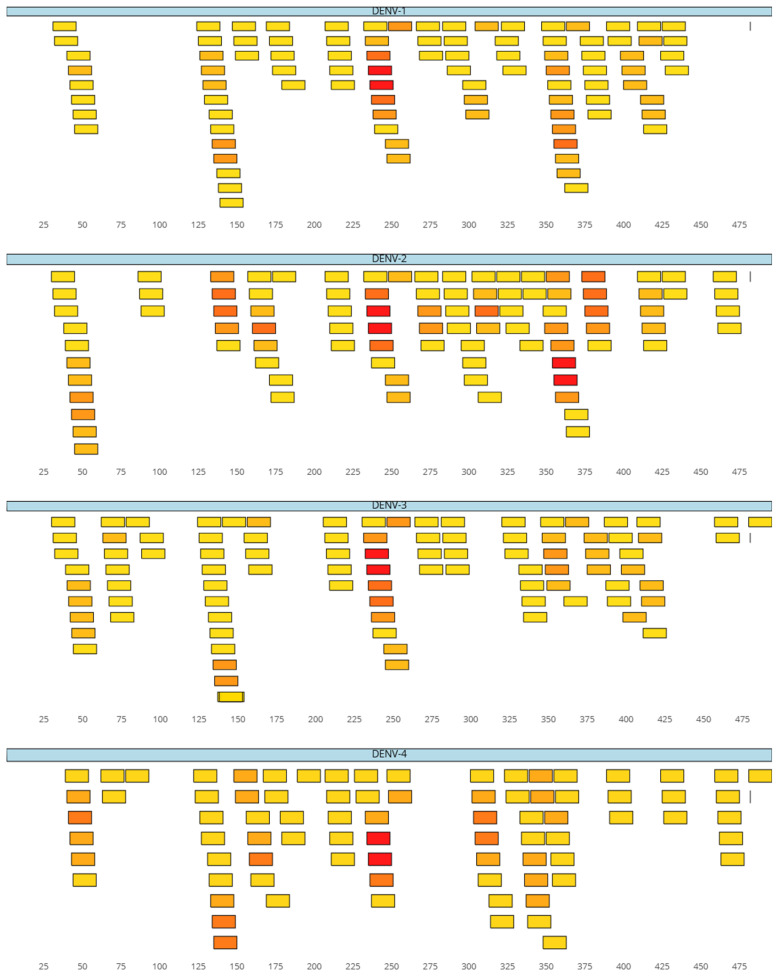
Location of predicted epitopes in E proteins from the four DENV serotypes for HLA-II. The E protein of each DENV serotype is displayed as a light blue bar, while predicted 15-mer epitopes are represented in a color gradient from yellow to red, indicating the number of HLA-II alleles to which each epitope can bind and ranging from 1 to 5 alleles for DENV-1, DENV-2, and DENV-3, and from 1 to 4 alleles for DENV-4. Regions in red thus indicate promiscuous epitopes, recognized by a greater number of alleles. Below each panel, the position of the amino acids in the polypeptide chain is reported.

Still regarding HLA-II alleles, it was observed that epitopes with high affinity for multiple alleles were frequently located in equivalent regions of the E protein of each DENV serotype (Figure 9). For DENV-1, the epitopes QDLLVTFKTAHAKKQ (234–248) and DLLVTFKTAHAKKQE (235–249) were able to bind to 5 alleles (DRB1*07:01/DRB1*15:01/DRB3*02:02/DRB4*01:01/DRB5*01:01 for both). For DENV-2, the epitopes QKETLVTFKNPHAKK (233–247), KETLVTFKNPHAKKQ (234–248), TVNPIVTEKDSPVNI (353–367), and VNPIVTEKDSPVNIE (354–368) were able to bind to 5 alleles (DRB1*07:01/DRB1*15:01/DRB3*02:02/DRB4*01:01/DRB5*01:01 for the first two and DRB1*03:01/DRB1*15:01/DRB3*01:01/DRB3*02:02/DRB4*01:01 for the last two). For DENV-3, the epitopes RKELLVTFKNAHAKK (231–245) and KELLVTFKNAHAKKQ (232–246) were able to bind to 5 alleles (DRB1*03:01/DRB1*15:01/DRB3*02:02/DRB4*01:01/DRB5*01:01 for the first one and DRB1*07:01/DRB1*15:01/DRB3*02:02/DRB4*01:01/DRB5*01:01 for the last one). For DENV-4, the epitopes YKERMVTFKVPHAKR (233–247) and KERMVTFKVPHAKRQ (234–248) were able to bind to 4 alleles (DRB1*15:01/DRB3*02:02/DRB4*01:01/DRB5*01:01 for both).

### 3.7. Conservation of Predicted Epitopes Among Circulating DENV Strains

Consensus E protein sequences generated from geographically diverse DENV isolates for each serotype exhibited high similarity to the corresponding reference sequences used throughout this study. Pairwise alignments revealed sequence identities of 97.8% for DENV-1, 98.4% for DENV-2, 99.4% for DENV-3, and 99.4% for DENV-4. Examination of the predicted immunologically relevant regions demonstrated complete conservation of all prominent B-cell epitopes as well as all promiscuous T-cell epitopes identified in the reference sequences (Figures S1–S4).

## 4. Discussion

Studies show that the E protein exhibits variations across DENV serotypes, potentially impacting its structural stability and interactions with the host immune system [18]. The sequence identity of this protein ranged from 63.08% to 77.69% among viral serotypes, with conservative substitutions predominating, followed by non-conservative and semi-conservative ones. Conservative substitutions likely maintain protein function, while non-conservative ones may affect protein conformation and antigenicity. Insertions and deletions were rare and likely represent isolated events. These variations are crucial for the immune response, contributing to differences in virulence and immunogenicity across serotypes and highlighting their significance for vaccine and therapy development [19].

Physicochemically, E proteins from the four DENV serotypes were dominated by non-polar residues, with polar residues slightly favoring neutral types in DENVs-1/3 and charged residues in DENVs-2/4. These features may influence membrane fusion and immune interactions [20]. Secondary structure predictions showed predominantly random coils across viral serotypes. DENVs-1/2 had more alpha-helices than beta-sheets, contrasting with literature on class II fusion proteins [21], while DENVs-3/4 displayed more β-sheets than alpha-helices, potentially conferring higher structural stability [22].

The E protein undergoes complex conformational rearrangements during receptor binding, endosomal trafficking, and membrane fusion [4]. Consequently, primary and secondary structure-based analyses provide only a partial view of the molecular determinants underlying viral entry and immune recognition. Integrative approaches involving three-dimensional structural modeling, molecular dynamics simulations, and protein–protein interaction analyses would offer a more comprehensive understanding of how the identified amino acid differences affect protein stability, receptor engagement, membrane fusion, and epitope exposure.

When it comes to post-translational modifications, distinct distributions of predicted consensus patterns were observed among the serotypes. For instance, CK2 phosphorylation sites were more frequent in DENVs-1/2 than DENVs-3/4, aligning with their roles in viral maturation and host interaction [23]. Glycosylation sites at N67 and N153 were present in all viral serotypes, likely affecting replication in mammalian and mosquito cells, besides impacting antibody recognition [24]. Additional glycosylation sites in DENV-3 (N470) and DENV-4 (N472) may alter immune evasion potential, although such a post-translational modification near the transmembrane domain may have limited accessibility and function [6]. Moreover, the unique TK phosphorylation sites predicted in DENV-4 represent potential regulatory features that warrant further investigation, with parallels to findings in Hepatitis C virus [25] and Ebola virus [26]. As both motifs—KEEQDQQY (83–90) and KMFESTY (400–406)—were fully preserved in the consensus sequence generated from geographically diverse contemporary DENV-4 isolates, they are not restricted to the selected reference sequence. However, it is important to emphasize that these findings reflect the presence of sequence motifs compatible with specific modifications and should not be interpreted as evidence that the corresponding biochemical modifications occur in vivo. Experimental approaches such as glycoproteomics, phosphoproteomics, or site-directed mutagenesis would be required to determine whether the predicted motifs are actually modified and contribute to viral replication or immune recognition.

Prediction of B-cell epitopes on E protein sequences from the four DENV serotypes revealed distinct patterns of antigenic propensity along the polypeptide chain, with regions of highest putative antigenicity distributed heterogeneously. One of these regions, located around residues D341 in DENV-1 and D339 in DENV-3, is consistent with the knowledge that E domain III (296–394), as an exposed site on the virion surface, is crucial for receptor recognition and viral neutralization [27]. The observed differences in predicted antigenic propensity among DENV serotypes may indicate variation in potential antibody recognition sites. Nevertheless, the present analysis was restricted to sequence-derived linear epitope prediction and therefore does not account for conformational epitopes formed by the tertiary and quaternary organization of the E protein on the virion surface. Moreover, hydrophilicity-based predictions alone cannot determine neutralizing capacity or the effectiveness of the immune response. Consequently, the biological relevance of these predicted antigenic regions and their contribution to antibody-mediated neutralization require experimental validation.

An important aspect of dengue immunobiology that was not addressed in the present study is the extensive cross-reactivity observed among DENV serotypes. Antibody responses generated during primary infection may recognize heterologous serotypes during subsequent infections, potentially contributing to either protection or antibody-dependent enhancement (ADE), depending on the specificity and neutralizing capacity of the antibodies involved [3]. Because the present analyses focused exclusively on sequence-derived biochemical and immunological predictions, they do not provide information regarding cross-reactive antibody responses, ADE, or the consequences of sequential infections.

The HLA-I alleles B*15:01/B*39:01 and the HLA-II alleles DRB1*15:01/DRB3*02:02/DRB4*01:01/DRB5*01:01 were predicted to bind epitopes of the four DENV serotypes, whereas the others showed some restriction. These findings suggest differences in the breadth of potential peptide presentation among HLA molecules, although their relationship to protective immunity or disease severity remains uncertain. Notably, alleles previously associated with either protection or susceptibility to severe dengue did not exhibit a consistent pattern of higher or lower predicted epitope recognition. For instance, HLA-I alleles, such as HLA-A*03 and HLA-A*24, are known to play distinct roles in immune responses to DENV serotypes: while the former is associated with a protective response and lower disease severity [28,29], the latter is linked to a weaker immune response, potentially favoring severe forms of dengue, such as DHF and DSS [30,31]. HLA-A*01, also associated with DHF, seems to be crucial in modulating susceptibility [32]. Regarding HLA-II alleles, HLA-DRB1*03:01 is associated with an effective immune response and protection against severe forms of disease [30]. In contrast, HLA-DRB1*07:01 shows ambiguous effects, being linked to both protection and susceptibility to DHF [33,34]. The DRB3, DRB4, and DRB5 loci also stand out, with implications in enhancing viral immunogenicity [35]. Furthermore, some HLA associations with disease severity seem to correlate with the DENV serotype inducing secondary infections [36]. While several HLA alleles included in this study have been previously associated with distinct clinical outcomes during DENV infection, the present analyses were not designed to establish correlations between predicted epitope abundance and disease severity. Therefore, the reported HLA-binding profiles should be interpreted as computational predictions of peptide presentation rather than indicators of clinical protection or susceptibility.

Although NetMHC-based algorithms are widely used and validated for epitope discovery, computational predictions may generate false-positive and false-negative results. Therefore, differences in epitope counts among serotypes are more appropriately viewed as indicators of distinct predicted binding landscapes rather than quantitative measures of relative immunogenicity, and the identified epitopes should be regarded as candidate targets for future experimental evaluation using peptide-binding assays and cellular immune response studies. It should also be noted that the present analysis employed representative HLA reference sets rather than population-specific allele frequencies. Consequently, the predicted epitope-binding profiles provide a comparative assessment of potential immune recognition but do not allow direct inference regarding immunogenicity at the population level. Because HLA allele frequencies vary substantially among geographic regions and ethnic groups, the relative contribution of individual epitopes may differ across human populations. Future studies incorporating population-specific HLA frequency data and larger panels of HLA alleles could provide a more comprehensive understanding of the potential immunogenicity of DENV E proteins in different epidemiological settings and improve the translational relevance of epitope prediction analyses.

Likewise, pre-existing immunity resulting from prior DENV exposure can substantially influence both humoral and cellular immune responses [37]. The present study did not evaluate immunological memory, T-cell cross-reactivity among serotypes, or the impact of previous infections on epitope recognition. Thus, the predicted HLA-binding profiles should be interpreted solely as indicators of potential peptide presentation rather than predictors of immune protection or disease outcome.

Another limitation of this study is that all analyses relied on a single reference sequence for each DENV serotype. While this strategy enabled a standardized comparison among serotypes, it does not capture the extensive intra-serotypic genetic diversity observed among circulating strains worldwide. To further evaluate the representativeness of the selected reference sequences, we assessed the conservation of the predicted epitopes using consensus sequences generated from recent DENV isolates originating from multiple continents. The high sequence identities observed between the consensus and reference sequences (97.8–99.4%), together with the complete conservation of all prominent B-cell epitopes and promiscuous T-cell epitopes, indicate that the major immunologically relevant regions identified in this study are preserved across geographically diverse viral populations. The observed conservation patterns strengthen the biological relevance of the comparative analyses performed herein and support the use of the selected reference sequences as representative models for each serotype, although rare variants with epitope-altering mutations cannot be excluded.

These findings highlight potential differences in biochemical and immunological features among DENV serotypes, which may impact disease progression. While the observations reported herein should be considered exploratory and predictive in nature, this research emphasizes the need for vaccines that account for these variations, improving efficacy and minimizing risks associated with ADE, while also underscoring the importance of future investigations to address viral genetic diversity and its implications for immunogenicity.

## Figures and Tables

**Table 1:** Molecular weight, net charge, and isoelectric point of E proteins from the four DENV serotypes.

Virus	Molecular Weight ^1^	Net Charge ^2^	Isoelectric Point
DENV-1	53.85	8.00	7.89
DENV-2	54.44	8.00	7.77
DENV-3	53.68	3.50	7.01
DENV-4	53.99	8.50	7.75

^1^ In kDa. ^2^ At pH 7.0.

## Data Availability

The original contributions presented in this study are included in the article/Supplementary Material. Further inquiries can be directed to the corresponding author.

## Supplementary Materials

The supporting information can be downloaded at https://www.mdpi.com/article/10.3390/cimb48060631/s1.
