# Supplementary material for: Serotype-Specific Biochemical and Immunological Signatures of Dengue Virus Envelope Proteins
Source: Curr Issues Mol Biol. 2026 Jun 17;48(6):631. doi: 10.3390/cimb48060631 (PMC13298711; doi:10.3390/cimb48060631)
Supplement: Supplementary file 1 [file cimb-48-00631-s001.zip › cimb-4342045-supplementary.pdf]

```
DENV-1_RefSeq      1 MRCVGIGNRDFVEGLSGATWVDVVLEHGSCVTTMAKDKPTLDIELLKTEV     50
                     |||||||||||||||||||||||||||||||||||||||||:||||||||
DENV-1_ConSeq      1 MRCVGIGNRDFVEGLSGATWVDVVLEHGSCVTTMAKNKPTLDIELLKTEV     50

DENV-1_RefSeq     51 TNPAVLRKLCIEAKISNTTTDSRCPTQGEATLVEEQDTNFVCRRTFVDRG    100
                     |||||||||||||||||||||||||||||||||||||.||||||||||||
DENV-1_ConSeq     51 TNPAVLRKLCIEAKISNTTTDSRCPTQGEATLVEEQDANFVCRRTFVDRG    100

DENV-1_RefSeq    101 WGNGCGLFGKGSLITCAKFKCVTKLEGKIVQYENLKYSVIVTVHTGDQHQ    150
                     |||||||||||||:||||||||||||||||||||||||||||||||||||
DENV-1_ConSeq    101 WGNGCGLFGKGSLLTCAKFKCVTKLEGKIVQYENLKYSVIVTVHTGDQHQ    150

DENV-1_RefSeq    151 VGNETTEHGTTATITPQAPTSEIQLTDYGALTLDCSPRTGLDFNEMVLLT    200
                     ||||||||||.|||||||||||||||||||||||||||||||||||||||
DENV-1_ConSeq    151 VGNETTEHGTIATITPQAPTSEIQLTDYGALTLDCSPRTGLDFNEMVLLT    200

DENV-1_RefSeq    201 MKKKSWLVHKQWFLDLPLPWTSGASTSQETWNRQDLLVTFKTAHAKKQEV    250
                     ||:|||||||||||||||||||||||||||||||||||||||||||||||
DENV-1_ConSeq    201 MKEKSWLVHKQWFLDLPLPWTSGASTSQETWNRQDLLVTFKTAHAKKQEV    250

DENV-1_RefSeq    251 VVLGSQEGAMHTALTGATEIQTSGTTTIFAGHLKCRLKMDKLILKGMSYV    300
                     ||||||||||||||||||||||||||||||||||||||||||.|||||||
DENV-1_ConSeq    251 VVLGSQEGAMHTALTGATEIQTSGTTTIFAGHLKCRLKMDKLTLKGMSYV    300

DENV-1_RefSeq    301 MCTGSFKLEKEVAETQHGTVLVQVKYEGTDAPCKIPFSSQDEKGVTQNGR    350
                     ||||||||||||||||||||||||||||||||||||||:|||||||||||
DENV-1_ConSeq    301 MCTGSFKLEKEVAETQHGTVLVQVKYEGTDAPCKIPFSTQDEKGVTQNGR    350

DENV-1_RefSeq    351 LITANPIVTDKEKPVNIEAEPPFGESYIVVGAGEKALKLSWFKKGSSIGK    400
                     ||||||||||||||||||.||||||||||:||||||||||||||||||||
DENV-1_ConSeq    351 LITANPIVTDKEKPVNIETEPPFGESYIVIGAGEKALKLSWFKKGSSIGK    400

DENV-1_RefSeq    401 MFEATARGARRMAILGDTAWDFGSIGGVFTSVGKLIHQIFGTAYGVLFSG    450
                     |||||||||||||||||||||||||||||||||||:||:|||||||||||
DENV-1_ConSeq    401 MFEATARGARRMAILGDTAWDFGSIGGVFTSVGKLVHQVFGTAYGVLFSG    450

DENV-1_RefSeq    451 VSWTMKIGIGILLTWLGLNSRSTSLSMTCIAVGMVTLYLGVMVQA    495
                     |||||||||||||||||||||||||||||||||||||||||||||
DENV-1_ConSeq    451 VSWTMKIGIGILLTWLGLNSRSTSLSMTCIAVGMVTLYLGVMVQA    495

| Perfect Match   : Strong Similarity   . Weak Similarity
  Prominent B-Cell Epitopes     Promiscuous T-Cell Epitopes
```

Figure S1. Pairwise alignment between the reference sequence (RefSeq) and the consensus sequence (ConSeq) generated from geographically diverse isolates of DENV-1 E protein. The most recent E protein sequences available from human blood isolates representing five continents were selected to generate a consensus sequence, which was subsequently aligned with the corresponding reference sequence to assess the conservation of the predicted prominent B-cell epitopes and promiscuous T-cell epitopes.

```
DENV-2_RefSeq      1 MRCIGMSNRDFVEGVSGGSWVDIVLEHGSCVTTMAKNKPTLDFELIKTEA     50
                     |||||:||||||||||||||||||||||||||||||||||||||||||||
DENV-2_ConSeq      1 MRCIGISNRDFVEGVSGGSWVDIVLEHGSCVTTMAKNKPTLDFELIKTEA     50

DENV-2_RefSeq     51 KQPATLRKYCIEAKLTNTTTESRCPTQGEPSLNEEQDKRFVCKHSMVDRG    100
                     ||||||||||||||||||||.|||||||||||||||||||||||||||||
DENV-2_ConSeq     51 KQPATLRKYCIEAKLTNTTTASRCPTQGEPSLNEEQDKRFVCKHSMVDRG    100

DENV-2_RefSeq    101 WGNGCGLFGKGGIVTCAMFRCKKNMEGKVVQPENLEYTIVITPHSGEEHA    150
                     |||||||||||||||||||.||||||||||||||||||||||||||||:|
DENV-2_ConSeq    101 WGNGCGLFGKGGIVTCAMFTCKKNMEGKVVQPENLEYTIVITPHSGEENA    150

DENV-2_RefSeq    151 VGNDTGKHGKEIKITPQSSITEAELTGYGTVTMECSPRTGLDFNEMVLLQ    200
                     ||||||||||||||||||||||||||||||||||||||||||||||||||
DENV-2_ConSeq    151 VGNDTGKHGKEIKITPQSSITEAELTGYGTVTMECSPRTGLDFNEMVLLQ    200

DENV-2_RefSeq    201 MENKAWLVHRQWFLDLPLPWLPGADTQGSNWIQKETLVTFKNPHAKKQDV    250
                     ||:|||||||||||||||||||||||||||||||||||||||||||||||
DENV-2_ConSeq    201 MEDKAWLVHRQWFLDLPLPWLPGADTQGSNWIQKETLVTFKNPHAKKQDV    250

DENV-2_RefSeq    251 VVLGSQEGAMHTALTGATEIQMSSGNLLFTGHLKCRLRMDKLQLKGMSYS    300
                     ||||||||||||||||||||||||||||||||||||||||||||||||||
DENV-2_ConSeq    251 VVLGSQEGAMHTALTGATEIQMSSGNLLFTGHLKCRLRMDKLQLKGMSYS    300

DENV-2_RefSeq    301 MCTGKFKVVKEIAETQHGTIVIRVQYEGDGSPCKIPFEIMDLEKRHVLGR    350
                     ||||||||||||||||||||||||||||||||||||||||||||||||||
DENV-2_ConSeq    301 MCTGKFKVVKEIAETQHGTIVIRVQYEGDGSPCKIPFEIMDLEKRHVLGR    350

DENV-2_RefSeq    351 LITVNPIVTEKDSPVNIEAEPPFGDSYIIIGVEPGQLKLNWFKKGSSIGQ    400
                     ||||||||||||||||||||||||||||||||||||||||||||||||||
DENV-2_ConSeq    351 LITVNPIVTEKDSPVNIEAEPPFGDSYIIIGVEPGQLKLNWFKKGSSIGQ    400

DENV-2_RefSeq    401 MFETTMRGAKRMAILGDTAWDFGSLGGVFTSIGKALHQVFGAIYGAAFSG    450
                     ||||||||||||||||||||||||||||||||||||||||||||||||||
DENV-2_ConSeq    401 MFETTMRGAKRMAILGDTAWDFGSLGGVFTSIGKALHQVFGAIYGAAFSG    450

DENV-2_RefSeq    451 VSWTMKILIGVIITWIGMNSRSTSLSVTLVLVGIVTLYLGVMVQA    495
                     |||||||||||:|||||||||||||||:|||||:|||||||||||
DENV-2_ConSeq    451 VSWTMKILIGVVITWIGMNSRSTSLSVSLVLVGVVTLYLGVMVQA    495

| Perfect Match   : Strong Similarity   . Weak Similarity
  Prominent B-Cell Epitopes      Promiscuous T-Cell Epitopes
```

Figure S2. Pairwise alignment between the reference sequence (RefSeq) and the consensus sequence (ConSeq) generated from geographically diverse isolates of DENV-2 E protein. The most recent E protein sequences available from human blood isolates representing five continents were selected to generate a consensus sequence, which was subsequently aligned with the corresponding reference sequence to assess the conservation of the predicted prominent B-cell epitopes and promiscuous T-cell epitopes.

```
DENV-3_RefSeq      1 MRCVGVGNRDFVEGLSGATWVDVVLEHGGCVTTMAKNKPTLDIELQKTEA     50
                     ||||||||||||||||||||||||||||||||||||||||||||||||||
DENV-3_ConSeq      1 MRCVGVGNRDFVEGLSGATWVDVVLEHGGCVTTMAKNKPTLDIELQKTEA     50

DENV-3_RefSeq     51 TQLATLRKLCIEGKITNITTDSRCPTQGEAVLPEEQDQNYVCKHTYVDRG    100
                     ||||||||||||||||||||||||||||||||||||||||||||||||||
DENV-3_ConSeq     51 TQLATLRKLCIEGKITNITTDSRCPTQGEAVLPEEQDQNYVCKHTYVDRG    100

DENV-3_RefSeq    101 WGNGCGLFGKGSLVTCAKFQCLEPIEGKVVQYENLKYTVIITVHTGDQHQ    150
                     |||||||||||||||||||||||||||||||:||||||||||||||||||
DENV-3_ConSeq    101 WGNGCGLFGKGSLVTCAKFQCLEPIEGKVVQHENLKYTVIITVHTGDQHQ    150

DENV-3_RefSeq    151 VGNETQGVTAEITPQASTTEAILPEYGTLGLECSPRTGLDFNEMILLTMK    200
                     ||||||||||||||||||||||||||||||||||||||||||||||||||
DENV-3_ConSeq    151 VGNETQGVTAEITPQASTTEAILPEYGTLGLECSPRTGLDFNEMILLTMK    200

DENV-3_RefSeq    201 NKAWMVHRQWFFDLPLPWASGATTETPTWNRKELLVTFKNAHAKKQEVVV    250
                     ||||||||||||||||||.|||||||||||||||||||||||||||||||
DENV-3_ConSeq    201 NKAWMVHRQWFFDLPLPWTSGATTETPTWNRKELLVTFKNAHAKKQEVVV    250

DENV-3_RefSeq    251 LGSQEGAMHTALTGATEIQNSGGTSIFAGHLKCRLKMDKLELKGMSYAMC    300
                     ||||||||||||||||||||||||||||||||||||||||||||||||||
DENV-3_ConSeq    251 LGSQEGAMHTALTGATEIQNSGGTSIFAGHLKCRLKMDKLELKGMSYAMC    300

DENV-3_RefSeq    301 TNTFVLKKEVSETQHGTILIKVEYKGEDAPCKIPFSTEDGQGKAHNGRLI    350
                     ||||||||||||||||||||||||||||||||||||||||||||||||||
DENV-3_ConSeq    301 TNTFVLKKEVSETQHGTILIKVEYKGEDAPCKIPFSTEDGQGKAHNGRLI    350

DENV-3_RefSeq    351 TANPVVTKKEEPVNIEAEPPFGESNIVIGIGDNALKINWYKKGSSIGKMF    400
                     ||||||||||||||||||||||||||||||||||||||||||||||||||
DENV-3_ConSeq    351 TANPVVTKKEEPVNIEAEPPFGESNIVIGIGDNALKINWYKKGSSIGKMF    400

DENV-3_RefSeq    401 EATERGARRMAILGDTAWDFGSVGGVLNSLGKMVHQIFGSAYTALFSGVS    450
                     |||.||||||||||||||||||||||||||||||||||||||||||||||
DENV-3_ConSeq    401 EATARGARRMAILGDTAWDFGSVGGVLNSLGKMVHQIFGSAYTALFSGVS    450

DENV-3_RefSeq    451 WVMKIGIGVLLTWIGLNSKNTSMSFSCIAIGIITLYLGAVVQA    493
                     |||||||||||||||||||||||||||||||||||||||||||
DENV-3_ConSeq    451 WVMKIGIGVLLTWIGLNSKNTSMSFSCIAIGIITLYLGAVVQA    493

| Perfect Match   : Strong Similarity   . Weak Similarity
  Prominent B-Cell Epitopes      Promiscuous T-Cell Epitopes
```

Figure S3. Pairwise alignment between the reference sequence (RefSeq) and the consensus sequence (ConSeq) generated from geographically diverse isolates of DENV-3 E protein. The most recent E protein sequences available from human blood isolates representing five continents were selected to generate a consensus sequence, which was subsequently aligned with the corresponding reference sequence to assess the conservation of the predicted prominent B-cell epitopes and promiscuous T-cell epitopes.

```
DENV-4_RefSeq      1 MRCVGVGNRDFVEGVSGGAWVDLVLEHGGCVTTMAQGKPTLDFELTKTTA      50
                     ||||||||||||||||||||||||||||||||||||||||||||||||||
DENV-4_ConSeq      1 MRCVGVGNRDFVEGVSGGAWVDLVLEHGGCVTTMAQGKPTLDFELTKTTA      50

DENV-4_RefSeq     51 KEVALLRTYCIEASISNITTATRCPTQGEPYLKEEQDQQYICRRDVVDRG     100
                     ||||||||||||||||||||||||||||||||||||||||||||||||||
DENV-4_ConSeq     51 KEVALLRTYCIEASISNITTATRCPTQGEPYLKEEQDQQYICRRDVVDRG     100

DENV-4_RefSeq    101 WGNGCGLFGKGGVVTCAKFSCSGKITGNLVQIENLEYTVVVTVHNGDTHA     150
                     ||||||||||||||||||||||||||||||||||||||||||||||||||
DENV-4_ConSeq    101 WGNGCGLFGKGGVVTCAKFSCSGKITGNLVQIENLEYTVVVTVHNGDTHA     150

DENV-4_RefSeq    151 VGNDTSNHGVTAMITPRSPSVEVKLPDYGELTLDCEPRSGIDFNEMILMK     200
                     ||||||||||||.|||||||||||||||||||||||||||||||||||||
DENV-4_ConSeq    151 VGNDTSNHGVTATITPRSPSVEVKLPDYGELTLDCEPRSGIDFNEMILMK     200

DENV-4_RefSeq    201 MKKKTWLVHKQWFLDLPLPWTAGADTSEVHWNYKERMVTFKVPHAKRQDV     250
                     ||||||||||||||||||||||||||||||||||||||||||||||||||
DENV-4_ConSeq    201 MKKKTWLVHKQWFLDLPLPWTAGADTSEVHWNYKERMVTFKVPHAKRQDV     250

DENV-4_RefSeq    251 TVLGSQEGAMHSALAGATEVDSGDGNHMFAGHLKCKVRMEKLRIKGMSYT     300
                     ||||||||||||||||||||||||||||||||||||||||||||||||||
DENV-4_ConSeq    251 TVLGSQEGAMHSALAGATEVDSGDGNHMFAGHLKCKVRMEKLRIKGMSYT     300

DENV-4_RefSeq    301 MCSGKFSIDKEMAETQHGTTVVKVKYEGAGAPCKVPIEIRDVNKEKVVGR     350
                     ||||||||||||||||||||||||||||||||||||||||||||||||||
DENV-4_ConSeq    301 MCSGKFSIDKEMAETQHGTTVVKVKYEGAGAPCKVPIEIRDVNKEKVVGR     350

DENV-4_RefSeq    351 IISSTPLAENTNSVTNIELEPPFGDSYIVIGVGNSALTLHWFRKGSSIGK     400
                     ||||||.||||||||||||||||||||||||||||:||||||||||||||
DENV-4_ConSeq    351 IISSTPFAENTNSVTNIELEPPFGDSYIVIGVGDSALTLHWFRKGSSIGK     400

DENV-4_RefSeq    401 MFESTYRGAKRMAILGETAWDFGSVGGLFTSLGKAVHQVFGSVYTTMFGG     450
                     ||||||||||||||||||||||||||||||||||||||||||||||||||
DENV-4_ConSeq    401 MFESTYRGAKRMAILGETAWDFGSVGGLFTSLGKAVHQVFGSVYTTMFGG     450

DENV-4_RefSeq    451 VSWMIRILIGFLVLWIGTNSRNTSMAMTCIAVGGITLFLGFTVQA    495
                     |||||||||||||||||||||||||||||||||||||||||||||
DENV-4_ConSeq    451 VSWMIRILIGFLVLWIGTNSRNTSMAMTCIAVGGITLFLGFTVQA    495

| Perfect Match   : Strong Similarity   . Weak Similarity
  Prominent B-Cell Epitopes     Promiscuous T-Cell Epitopes
```

Figure S4. Pairwise alignment between the reference sequence (RefSeq) and the consensus sequence (ConSeq) generated from geographically diverse isolates of DENV-4 E protein. The most recent E protein sequences available from human blood isolates representing five continents were selected to generate a consensus sequence, which was subsequently aligned with the corresponding reference sequence to assess the conservation of the predicted prominent B-cell epitopes and promiscuous T-cell epitopes.
